# PIWI-interacting RNAs: who, what, when, where, why, and how

**DOI:** 10.1038/s44318-024-00253-8

**Published:** 2024-09-26

**Authors:** Astrid D Haase, Rene F Ketting, Eric C Lai, Ronald P van Rij, Mikiko Siomi, Petr Svoboda, Josien C van Wolfswinkel, Pei-Hsuan Wu

**Affiliations:** 1 National Institutes of Diabetes and Digestive and Kidney Diseases, National Institutes of Health, Bethesda, MD USA; 2 Biology of Non-coding RNA Group, Institute of Molecular Biology, Mainz, Germany; 3 Developmental Biology Program, Sloan Kettering Institute, New York, USA; 4 Department of Medical Microbiology, Radboud University Medical Center, Nijmegen, The Netherlands; 5 Department of Biological Sciences, Graduate School of Science, The University of Tokyo, Tokyo, Japan; 6 Institute of Molecular Genetics of the Czech Academy of Sciences, Prague, Czech Republic; 7 Department of Molecular Cellular and Developmental Biology, Yale University, New Haven, CT USA; 8 Department of Genetic Medicine and Development, University of Geneva, Geneva, Switzerland

**Keywords:** RNA Biology

## Abstract

This commentary highlights, from an interdisciplinary perspective, recent advances and key outstanding questions in the field of piRNA biology.

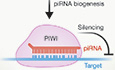

## Teaser

PIWI-interacting RNAs (piRNA) suppress selfish genetic elements and are essential for germ cell biology in animals. They also play critical roles in regeneration in planaria, regulate gene expression in adult mammalian testes, and participate in antiviral defense in mosquitoes. Inspired by a recent workshop on PIWI proteins and piRNAs, this commentary aims to summarize fundamental aspects of piRNA biology, highlight recent advances, and discuss key outstanding questions. It is written by and for biochemists, geneticists, and evolutionary biologists, and represents our interdisciplinary perspective.

Argonaute proteins and their small guide RNAs play crucial roles in genome defense and gene silencing across all branches of life. In animals, three classes of small RNAs—small interfering RNAs (siRNAs), microRNAs (miRNAs), and piRNAs—can be distinguished by their specific RNA precursors and associated Argonaute proteins (Onishi et al, 2021; Ozata et al, 2019). piRNAs are bound to members of the PIWI-subfamily of Argonaute proteins, which are primarily expressed in germ cells, and mediate transcriptional or post-transcriptional silencing of complementary target RNAs (Aravin et al, 2007; Czech et al, 2018).

piRNAs comprise millions of diverse, non-conserved sequences, most of which have not been assigned a function (Genzor et al, 2021; Wu and Zamore, 2021). Identification of piRNAs requires the verification of PIWI protein expression, which is not ubiquitous, purification of PIWI-piRNA complexes, and sequencing of the associated piRNAs. Annotation of short RNA fragments solely using databases results in false positives. Finally, genetic follow-up studies are essential to elucidate piRNA functions (Choi et al, 2021; Gebert et al, 2021; Wu et al, 2020).

**Figure 1:**
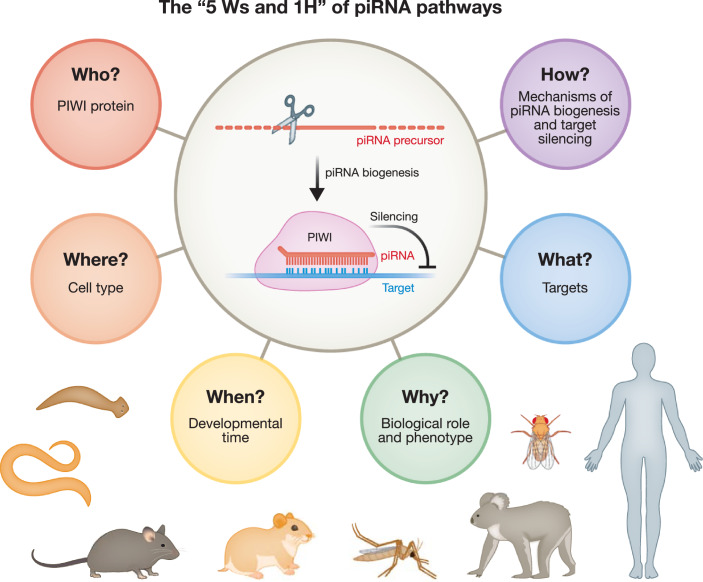
The ‘Ws and H’ guidelines for piRNA biology in animals. At the core of all piRNA pathways are piRNA silencing complexes (piRISCs) that minimally consist of a PIWI protein and its associated piRNA. In animals, piRNAs are generated from single-stranded precursors by different processing enzymes. Mature piRISCs recognize target RNAs with sequence complementarity in the nucleus or in the cytoplasm and induce transcriptional or post-transcriptional silencing, respectively. piRNA pathways serve different functions in different cell types and organisms. The identity of the PIWI protein partner (WHO?), the specific cell type (WHERE?), the developmental stage (WHEN?), the biological role -best illustrated by loss-of-function phenotypes of a specific PIWI gene or piRNA-producing locus- (WHY?), functional targets (WHAT?), and the mechanisms through which piRISCs form and act (HOW?) define distinct piRNA pathways.

In exploring piRNA biology, we encourage new travelers to consider the ‘5 Ws and 1 H’ of piRNA pathways (Fig. 1): Who is the PIWI protein partner? Where, when, and why are these PIWI-piRNA complexes required? How are they made and what are their targets? The identity of the PIWI protein, its expression pattern, piRNA repertoire, and other attributes specify piRNA pathways, facilitate conversations, and may be considered for a more formal subclassification in the future. This collaborative commentary offers a glimpse into the diverse world of piRNA biology across different organisms and aims to inspire further reading and discussion.

## piRNA pathways in different species: genome defense, gene regulation, and more

Genome defense against selfish genetic elements is the conserved function of piRNAs in animals, and rapid evolution of piRNA precursors illustrates ongoing conflict between selfish genetic elements and host genomes (Aravin et al, 2007; Onishi et al, 2021; Ozata et al, 2019). Germ cells carry immortal genomes, preserve a species’ genomic identity, and are at the center of evolutionary change. Selfish genetic elements that successfully invade germline genomes become an integral part of future generations.

Although transposable elements and viruses are the most famous selfish genetic elements, genomes also harbor endogenous selfish genes termed “meiotic drive” loci (Lai and Vogan, 2023). As these genes manipulate gametogenesis to promote their own transmission in violation of Mendel’s laws, their wild-type activities can be deleterious. Several meiotic drivers distort progeny sex ratios and thus impose strong pressure to innovate genetic suppressors. siRNAs neutralize different meiotic drivers and thereby preserve species in insects, and contributions of piRNAs are emerging (Chen et al, 2021; Vedanayagam et al, 2023). This suggests that meiotic drive genes, which were historically recognized *via* fortuitous genetic anomalies, might be identifiable *via* signatures of targeting by piRNAs or siRNAs.

In insects, piRNA pathway genes are fast-evolving and the PIWI gene family underwent frequent duplications and eliminations. While piRNA pathways are restricted to gonads in several widely studied model organisms, including *Drosophila melanogaster*, piRNA pathways also operate in somatic cells in arthropods (Lewis et al, 2018). In the yellow fever mosquito *Aedes aegypti*, four PIWI genes are expressed in both soma and germline (Miesen et al, 2016). In addition to transposons, which remain the major source of piRNAs, non-retroviral RNA viruses are processed into and are cleaved by somatic piRNAs, suggesting functions in antiviral defense with heritable properties (Miesen et al, 2016; Suzuki et al, 2020). Further exploration of the piRNA pathway in animals with an expanded PIWI gene family will provide a broader perspective of piRNA functions and mechanisms and potentially lead to new practical applications.

Highly regenerative animals, such as planarians, express PIWI proteins and piRNAs in their germ cells as well as in adult pluripotent stem cells and require the piRNA pathway for regeneration (van Wolfswinkel, 2014). Recent studies of the adult pluripotent cells (known as neoblasts) in the planarian *Schmidtea mediterranea* uncovered a role for PIWI-piRNA complexes in regulating gene expression required for neoblast maintenance (Allikka Parambil et al, 2024). Furthermore, PIWI-piRNA complexes are essential during cell differentiation by preventing the opening of chromatin at repetitive regions in proximity to activated tissue-specific genes (Li et al, 2021). Studies of the piRNA pathway in this stem cell system will expand our appreciation of the developmental importance of piRNA pathways.

Knowledge about mammalian piRNAs has largely been inferred from mice, which operate and require distinct piRNA pathways in fetal and adult testes to maintain fertility (Aravin et al, 2007; Onishi et al, 2021; Ozata et al, 2019). In fetal testes, pre-pachytene piRNAs in nuclear PIWIL4(MIWI2)-piRNA complexes establish DNA and histone modifications on transposons that ensure their transcriptional repression. The initiation of meiosis jumpstarts a second piRNA pathway executed by cytoplasmic piRNAs associated with PIWIL1(MIWI) and PIWIL2(MILI): the pachytene piRNA pathway. Despite diverse sequences, disrupted pachytene piRNA biogenesis impairs spermatogenesis in mice, hamsters, and humans alike. However, most piRNAs do not seem to target anything and only a few target-RNA pairs have been identified (Choi et al, 2021; Reuter et al, 2011; Wu et al, 2020). Accumulation of structural evidence and in vivo interrogation of target cleavage confirmed a long-suspected theory—that pachytene piRNAs do not obey the miRNA or siRNA targeting rules and allow significantly more flexibility (Anzelon et al, 2021; Gainetdinov et al, 2023; van Wolfswinkel, 2024; Vourekas et al, 2012). This has important implications for the quest to identify authentic targets. Whether nuclear pre-pachytene piRISCs employ the same targeting rules is unclear. Results from piRNA-targeting studies are bound to further our understanding of the excessive piRNA sequence diversity and identify functionally redundant sequences and those without any function.

More than two decades after their discovery, we have only just begun to accumulate sufficient data and resources to interrogate PIWI-piRNA pathways in other mammals including humans (Loubalova et al, 2023). It has long been puzzling that mutations in essential piRNA pathway genes result in male sterility but do not affect female fertility in mice. The discovery of an alternative siRNA pathway in murine oocytes started to shed light on this sex-based variation (Flemr et al, 2013). Humans, golden hamsters, and a growing list of mammals use an essential ovary-specific PIWIL3-piRNA pathway to regulate female fertility (Hasuwa et al, 2021; Loubalova et al, 2021; Roovers et al, 2015; Williams et al, 2015; Zhang et al, 2021). This example illustrates that different small RNA pathways can collaborate or even substitute for each other on specific occasions and highlights the need for studies in additional non-model animals.

## Molecular mechanism of piRNA biogenesis

piRNAs guard genome integrity already in unicellular eukaryotes. In ciliates, piRNA pathways facilitate genome elimination to rid somatic genomes of mobile genetic elements. A major difference between ciliate and animal piRNA biogenesis is that most ciliate piRNAs are generated from double-stranded RNAs using Dicer whereas animals capitalize on single-stranded RNA substrates to produce mature piRNAs. Why animals favor single-stranded RNA precursors that require different processing mechanisms is a question awaiting an answer.

Fly and mouse piRNAs are generated from RNA precursors by the ZUC endonuclease (Zucchini (ZUC) in *Drosophila*, PLD6(MitoPLD) in mice) or by coordinated slicing during ping-pong, which is the amplification of piRNAs pairs by the intrinsic nuclease activity of PIWI proteins (Czech et al, 2018; Loubalova et al, 2023; Onishi et al, 2021; Ozata et al, 2019). A two-step mechanism combining cleavage preferences of the ZUC processor complex and binding preferences by PIWI proteins enriches for uracil in the first position of mature piRNAs in these animals (Stein et al, 2019). Interestingly, a different endoribonuclease called ‘precursor of 21U RNA 5′-end cleavage holoenzyme’ (PUCH) always leaves a uracil in the first position in *C. elegans* (Ketting and Cochella, 2021; Podvalnaya et al, 2023). Why two unrelated processing mechanisms converge on a preference for uracil in the first position of mature piRNAs remains unknown.

While previous studies have established a framework for mechanisms of piRNA biogenesis and function, we are just beginning to understand how these mechanisms are regulated (Onishi et al, 2021). Transcription factors required for the coordinated expression of pachytene piRNA pathway genes and their piRNA precursors have been identified in mice (Li et al, 2013; Zhou et al, 2017), and current efforts are zooming in on master transcription factors of *Drosophila* piRNA programs. PIWI’s intrinsic slicer activity is enhanced by the small zinc-finger protein GTSF1 and its paralogs, and cytoplasmic PIWI-piRNA complexes require GTSF1 to effectively cleave complementary targets (Arif et al, 2022). Additional regulators that positively or negatively impact PIWI’s slicer activity are starting to emerge. Finally, ping-pong amplification of piRNA pairs is pervasive in *Drosophila* germ cells, requires specific co-factors including the RNA helicase DDX4(Vasa) and Tudor proteins, and occurs in germ granules (Siomi et al, 2010; Xiol et al, 2014). In contrast to germ cells, ping-pong is inhibited in ovarian somatic cells through a mechanism that involves lethal(3)malignant brain tumor (l(3)mbt) (Yamamoto-Matsuda et al, 2022). Similarly, ping-pong is essential to fuel the nuclear PIWI protein PIWIL4(MIWI2) in murine gonocytes but seems to be inhibited by the Tudor protein RNF17 in meiotic spermatocytes (Wasik et al, 2015). The regulators, points of regulation, and molecular mechanisms might be specific to individual piRNA pathways, cell types, or species, but the necessity to tightly manage piRNA programs emerges as a conserved theme and inspires future studies.

The molecular mechanisms of piRNA pathways are getting resolved at a rapid pace. An important step now is to connect these pathways to the canonical RNA and chromatin machineries. This is not equivalent to ‘figuring out the nitty gritty details’ but an extremely important process for a number of reasons. First, it enhances the mechanistic understanding of the piRNA systems and will no doubt reveal where our models are simply still incorrect. Second, it will show how novel regulatory modules can be coupled to basic machineries such as transcription, RNA export, and RNA decay as well as processes affecting subcellular localization and RNA-protein granules. Third, particularly when this is done in various organisms, it will show the flexibility of gene regulatory systems and possibly how, when, and why piRNA systems started to diverge from each other. Our efforts to understand molecular mechanisms of piRNA pathways are stimulated by novel protein structures and the availability of AI-based structural predictions. Elucidating mechanisms of piRNA pathways and connections to general cellular machines is not only interesting for the piRNA field but will also be important for the mechanistic understanding of basic processes like transcription, RNA turnover, and granule formation.

## Conclusions

piRNA pathways are as fascinating as they are diverse. They are essential for fertility in most and stem cell biology in some animals, contribute to the regulation of gene expression, and help defend against viruses in some instances. On our interdisciplinary journey, we have learned much about these pathways from established model organisms. However, fast-evolving variations promise more mechanisms and biological insight to be found in non-standard model organisms and through comparative biological studies. Finally, understanding mechanisms of piRNA biogenesis and function holds great potential for the development of novel biotechnology and RNA-guided epigenetic therapy.

## Glossary

Argonaute proteins: are defined by a PIWI and a PAZ domain and participate in different small RNA-guided silencing pathways in all branches of life.
PIWI proteins: are a subfamily of Argonaute proteins. Their expression is largely restricted to germ cells in animals.
PIWI-interacting RNAs (piRNAs): are small RNAs that interact with PIWI proteins.
Pre-pachytene piRNAs: are associated with PIWIL2 and PIWIL4 in gonocytes (early spermatogonial stem cells) in mammals.
Pachytene piRNAs: are associated with PIWIL1 and PIWIL2 during the pachytene stage of meiosis and specific to mammalian spermatogenesis.
Ping-pong: is the amplification of piRNA pairs occurring when cytoplasmic PIWI-piRNA complexes slice a target RNA and the 5′ mono-phosphorylated end of the 3′ cleavage product is loaded into another PIWI protein to become a secondary piRNA.
